# Contour integration deficits at high spatial frequencies in children treated for anisometropic amblyopia

**DOI:** 10.3389/fnins.2023.1160853

**Published:** 2023-07-26

**Authors:** Shu-Qi Jiang, Yan-Ru Chen, Xiang-Yun Liu, Jun-Yun Zhang

**Affiliations:** ^1^School of Psychological and Cognitive Sciences and Beijing Key Laboratory of Behavior and Mental Health, Peking University, Beijing, China; ^2^The Affiliated Tengzhou Hospital of Xuzhou Medical University, Tengzhou, Shandong, China

**Keywords:** amblyopia, anisometropia, contour integration, spatial vision, global processing

## Abstract

**Purpose:**

This study was conducted to reexamine the question of whether children treated for anisometropic amblyopia have contour integration deficits. To do so, we used psychophysical methods that require global contour processing while minimizing the influence of low-level deficits: visibility, shape perception, and positional uncertainty.

**Methods:**

Thirteen children with anisometropic amblyopia (age: 10.1 ± 1.8 years) and thirteen visually normal children (age: 10.8 ± 2.0 years) participated in this study. The stimuli were closed figures made up of Gabor patches either in noise or on a blank field. The contrast thresholds to detect a circular contour on a blank field, as well as the thresholds of aspect ratio and contour element number to discriminate a circular or elliptical contour in noise, were measured at Gabor spatial frequencies of 1.5, 3, and 6 cpd for amblyopic eyes (AEs), fellow eyes (FEs), and normal control eyes. Visual acuities and contrast sensitivity functions for AEs and FEs and the Randot stereoacuity were measured before testing.

**Results:**

The AEs showed contrast deficits and degraded shape perception compared to the FEs at higher spatial frequencies (6 cpd). When the influence of abnormal contrast sensitivity and shape perception were minimized, the AEs showed contour integration deficits at spatial frequencies 3 and 6 cpd. These deficits were not related to basic losses in contrast sensitivity and acuity, stereoacuity, and visual crowding. Besides, no significant difference was found between the fellow eyes of the amblyopic children and the normal control eyes in the performance of contour integration.

**Conclusion:**

After eliminating or compensating for the low-level deficits, children treated for anisometropic amblyopia still show contour integration deficits, primarily at higher spatial frequencies, which might reflect the deficits in global processing caused by amblyopia. Contour integration deficits are likely independent of spatial vision deficits. Refractive correction and/or occlusion therapies may not be sufficient to fully restore contour integration deficits, which indicates the need for the development of clinical treatments to recover these deficits.

## Introduction

Amblyopia is a developmental visual disorder due to the degradation of the retinal image that results from strabismus, anisometropia, or deprivation in early childhood (Holmes and Clarke, 2006; Levi, 2013). It is accompanied by complex neural deficits in both the striate and extrastriate cortex (Kiorpes et al., 1998; Barnes et al., 2001; Conner et al., 2007; El-Shamayleh et al., 2010; Bi et al., 2011; Tao et al., 2014; Shooner et al., 2015; Wang et al., 2017), even in subcortical pathways (Wen et al., 2021). Amblyopia is a major cause of unilateral visual loss, especially in children (Birch, 2013). It impairs a range of visual functions, such as decreased visual acuity (Birch and Swanson, 2000; Levi et al., 2011), reduced spatial contrast sensitivity (Hess and Howell, 1977; Levi and Harwerth, 1977; Bradley and Freeman, 1981), impaired stereopsis (McKee et al., 2003; Giaschi et al., 2013), increased positional uncertainty (Levi and Klein, 1986; Hess and Holliday, 1992; Demanins and Hess, 1996), and abnormal global shape perception (Hess et al., 1999; Dallala et al., 2010). So far, the most commonly used treatments for amblyopia include occlusion therapy and optical correction, both of which have been shown to improve visual acuity (Moseley et al., 2002; Clarke et al., 2003; Holmes et al., 2003; Repka et al., 2003; Stewart et al., 2004; Awan et al., 2005) and can also improve stereoacuity to a certain extent (Lee and Isenberg, 2003; Wallace et al., 2011). However, neither of them was sufficient to completely resolve the impairments in visual functions caused by amblyopia (Levi, 2020).

Contour integration is the process of integrating local fragments across the visual field into paths or shapes and it plays an important role in the perception of natural images in daily visual experience (Hamm et al., 2014). Using a “snake-like” contour path of Gabors embedded in a noise background, Field et al. (1993) found that the continuity of neighboring contour elements played a primary role in the detection of contours and proposed that the long-range horizontal interactions between V1 neurons constitute the underlying mechanisms for contour integration, which was confirmed in later neurophysiological studies (Kapadia et al., 1995, 2000; Bauer and Heinze, 2002; Li et al., 2006; Gilad et al., 2013). On the other hand, some neuroimaging studies showed that both the striate and extrastriate cortex were involved in contour integration (Altmann et al., 2003; Kourtzi et al., 2003; Kuai et al., 2017). Moreover, accumulating evidence suggests that the feedback loops from higher to lower visual areas played an important role in contour integration (Chen et al., 2014; Mijovic et al., 2014; Liang et al., 2017; Li et al., 2019).

Previous studies on contour integration in amblyopia mainly assessed adults with amblyopia and found impairments in contour integration in their amblyopic eyes (AEs) with different tests (Hess et al., 1997; Kovács et al., 2000; Mussap and Levi, 2000; Levi et al., 2007). Psychophysical deficits in contour detection in the noisy image are consistently observed in strabismic amblyopia (Hess and Howell, 1977; Hess et al., 1997, 1999; Kovács et al., 2000; Levi et al., 2007), but not always in anisometropic amblyopia (Hess and Demanins, 1998; Levi et al., 2007). For example, Hess and Demanins (1998) found no contour integration deficits in most adults with anisometropic amblyopia with a contour detection task. Yet, Levi et al. (2007) found a mild degree of genuine contour integration deficits in adults with anisometropic amblyopia with a contour discrimination test. These contradictory findings suggest that different contour integration tests may produce inconsistent results. Likely, the task in Hess and Demanins (1998) was not sensitive enough to detect the deficits in anisometropic amblyopia.

On the other hand, as spatial integration develops throughout childhood and matures late (Kovacs et al., 1999; Hadad et al., 2010), contour integration could be affected differently in amblyopic children and adults. So far, only a few studies examined contour integration in amblyopic children and they were based on a single task (Chandna et al., 2001, 2004). Chandna et al. (2001) used a contour detection test and reported deficits in most children with newly diagnosed anisometropic amblyopia at a Gabor spatial frequency of 5 cpd (cycles per degree). Chandna et al. (2004) further reported that contour detection deficits in amblyopic children could almost recover with 8 weeks of treatment (refractive correction alone or in combination with occlusion therapy). The recovery of contour integration deficits was even more significant than the recovery of visual acuity. The authors explained that contour integration could be less severely disrupted and retain a greater degree of plasticity due to its longer developmental period and late maturation (Chandna et al., 2004).

However, if contour integration deficits in amblyopic children could recover with conventional treatments, one could reasonably assume that adults with amblyopia who were treated at an early age would no longer have impairments in contour integration. Yet, this is not the case as mentioned before. We note that the test Chandna et al. (2004) used was based on a limited number of cards, which might not be as accurate as computer-based tests in detecting deficits in amblyopia, especially in those who have received treatments whose deficits might be less severe. Moreover, there is evidence indicating visual function impairments in the fellow eyes (FEs) compared to normal control eyes (Meier and Giaschi, 2017; Birch et al., 2019), including in contour integration in adult amblyopia (Kovács et al., 2000). Thus, the findings of Chandna et al. (2004), which were based solely on interocular differences without comparisons with normal control eyes, might not be sufficient to support the conclusion that contour integration impairments could be corrected by refractive correction and/or occlusion therapy. Also, since the losses in visual functions caused by amblyopia are mainly at middle and high spatial frequencies (Levi, 2013), contour integration deficits may vary at different spatial frequencies for amblyopic children. Taken together, it is necessary to further investigate contour integration at different spatial frequencies in amblyopic children who have had conventional treatments using more rigorous methods.

The current study aimed to comprehensively investigate contour integration deficits in children treated for anisometropic amblyopia. We adopted contour tasks from Levi et al. (2007) to systematically evaluate the performance in contour integration at spatial frequencies of 1.5, 3, and 6 cpd in children with anisometropic amblyopia and a group of age-matched controls. In separate experiments, we measured the threshold of contour contrast detection and shape perception. After compensating for the low-level deficits of decreased contrast sensitivity and degraded shape perception in the AEs, we measured the threshold in a contour discrimination task. Moreover, we investigated the relationship between contour integration performance and visual functions, such as contrast sensitivity function, visual acuity, stereoacuity. These results might help us to create a more comprehensive understanding of contour integration deficits in amblyopic children and to develop possible treatments as well.

## Methods

### Participants

Thirteen children with unilateral anisometropic amblyopia (age range, 8.0–12.9 years, mean ± *SD* = 10.1 ± 1.8 years, 9 boys and 4 girls) participated in this study. The other thirteen children aged 8 to 13 years (4 boys and 9 girls, mean ± *SD* = 10.8 ± 2.0 years) with normal or correct-to-normal visual acuity and normal steroacuity (mean ± *SD* = 31.54 ± 10.49 arcsec) also participated in this study as a control group. Each observer’s vision was best corrected with a tumbling E acuity chart at the designated viewing distance of 5 meters. Testing was performed with the observers wearing the best optical correction, and the visual acuity values reported throughout the paper were for best-corrected acuity.

All amblyopic observers had ophthalmological examinations, and detailed clinical information was given in Table 1. Amblyopia was defined as a difference in best-corrected visual acuity of two or more logMAR lines between the two eyes with better acuity in the fellow eye (FE). Anisometropia was defined as ≥1.50 D difference between eyes in spherical power or ≥1.00 D difference between eyes in cylindrical power in any meridian. All amblyopic observers had received refractive correction and/or occlusion therapy, starting at the age of 7.5 ± 2.9 years, with a treatment length ranging from 2 to 73.1 months. Their visual acuity had improved by 0.32 ± 0.22 log units on a logarithmic visual acuity chart after treatment. The study adhered to the tenets of the Declaration of Helsinki and was approved by the ethics committees of Tengzhou Central People’s Hospital and Peking University. Informed consent was obtained from each observer’s parent or guardian after an explanation of the nature and possible consequences of the study.

**Table 1:** The characteristics of children with anisometropic amblyopia.

Observer	Age (years)	Gender	Eye	Correction	Visual Acuity Pre-experiment (LogMAR)	Stereoacuity (arcsec)	Treatment	Type	Starting age	Starting acuity	Length (months)
S1	12.1	M	AE (L)	+5.50/−1.00 × 10	0.3	30	Patch and glasses	7.5	0.52	9.2
			FE (R)	−2.50	−0.08				0	
S2	8.3	M	AE (R)	+5.25/−0.75 × 152	0.1	20	Patch and glasses	3	0.4	73.1
			FE (L)	+3.50/−1.25 × 8	−0.08				0.22	
S3	10.1	F	AE (L)	+2.00	0.1	20	Patch and glasses	7.1	0.4	36.6
			FE (R)	Plano	−0.18				0	
S4	12.3	F	AE (L)	+2.00	0.22	200	Patch and glasses	9.8	0.7	29.5
			FE (R)	Plano	0				0	
S5	8	M	AE (L)	+3.25/−0.50 × 25	0.1	70	Glasses	6	0.52	18.5
			FE (R)	Plano	−0.08				0	
S6	9.9	M	AE (R)	+7.00	0.22	40	Glasses	4.1	0.92	71.3
			FE (L)	+7.50/−1.25 × 160	0				0.82	
S7	8.6	M	AE (L)	+6.50	0.4	200	Patch and glasses	7.9	1	8.4
			FE (R)	Plano	−0.08				0	
S8	8.4	M	AE (R)	+5.00/−0.25 × 115	0.3	F	Patch and glasses	7.5	0.3	13.8
			FE (L)	+1.50/−0.75 × 175	0				0	
S9	8.9	M	AE (L)	+6.50/−1.00 × 175	0.22	F	Patch and glasses	3	0.6	72.5
			FE (R)	+2.75/−0.25 × 155	0				0.22	
S10	12.9	M	AE (R)	+3.50/−0.75 × 5	0.4	F	Patch and glasses	12.7	0.4	2
			FE (L)	−0.50/−0.75 × 170	0				0	
S11	11.9	M	AE (R)	+6.00/−1.25 × 176	0.52	F	Patch and glasses	10.8	0.82	12.2
			FE (L)	+1.75/−0.50 × 167	0				0	
S12	9	F	AE (R)	+4.50/−1.25 × 170	0.82	F	Patch and glasses	8.8	0.82	2.7
			FE (L)	Plano	0				0	
S13	11	F	AE (R)	+5.00/−1.00 × 135	0.4	70	Patch and glasses	9	0.82	12.4
			FE (L)	−1.25	0				0	

All children with anisometropic amblyopia had received conventional treatments of occlusion therapy and/or refractive correction. Gender: F, female; M, male. Eye: AE, amblyopic eye; FE, fellow eye; R, right; L, left. Stereoacuity: F, failed (unable to see stereopsis at the largest test disparity 500 arcsec).

### Apparatus and stimuli

The stimuli were generated with MATLAB-based Psychtoolbox-3 (Pelli, 1997) and presented on a 21-in. Sony G520 CRT monitor with a display resolution of 1,024 × 768 pixels and a frame rate of 60 Hz. The luminance of the monitor was linearized by an 8-bit look-up table (58.2 cd/m^2^ mean luminance). Observers viewed the displays monocularly with the non-tested eye patched. The normal controls were measured in one eye which was randomly selected before testing (7 observers used their dominant eyes and the other 6 observers used their non-dominant eyes). A chin-and-head rest help stabilize the head of the observer. The experiments were run in a dimly lit room.

**Figure 1:**
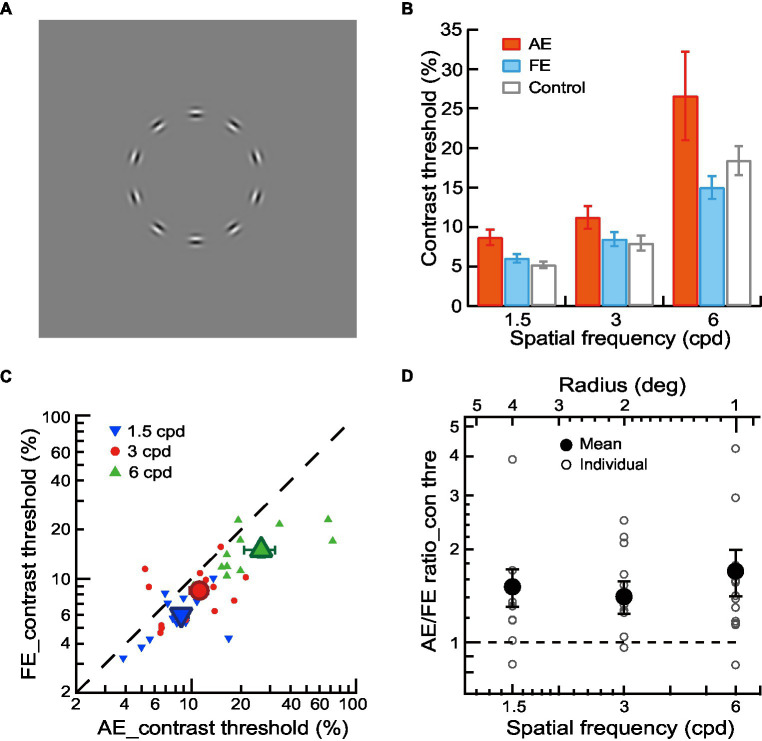
Contrast detection of contour in amblyopic children and children with normal vision. **(A)** Circular contours in a blank field were used for contour detection experiments. **(B)** Mean contrast thresholds of AEs (red bars), FEs (blue bars), and normal control eyes (gray bars) across different spatial frequencies. **(C)** Contrast thresholds of FEs and AEs. Data points below the diagonal line indicate a trend of higher thresholds in AEs than in FEs. Gabor spatial frequency is coded by different symbols and colors. The larger dots show mean thresholds and the smaller ones show individuals’ data for each amblyopic observer. **(D)** The AE/FE ratio of contrast threshold as a function of Gabor spatial frequency (lower abscissa). The radius (top abscissa) decreased proportionally as the Gabor spatial frequency increased. Data points above the dashed line indicate higher thresholds in the AEs than the FEs. The larger dots show mean thresholds and the smaller ones show individuals’ data for each amblyopic observer. Error bars indicate one standard error of the mean.

**Figure 2:**
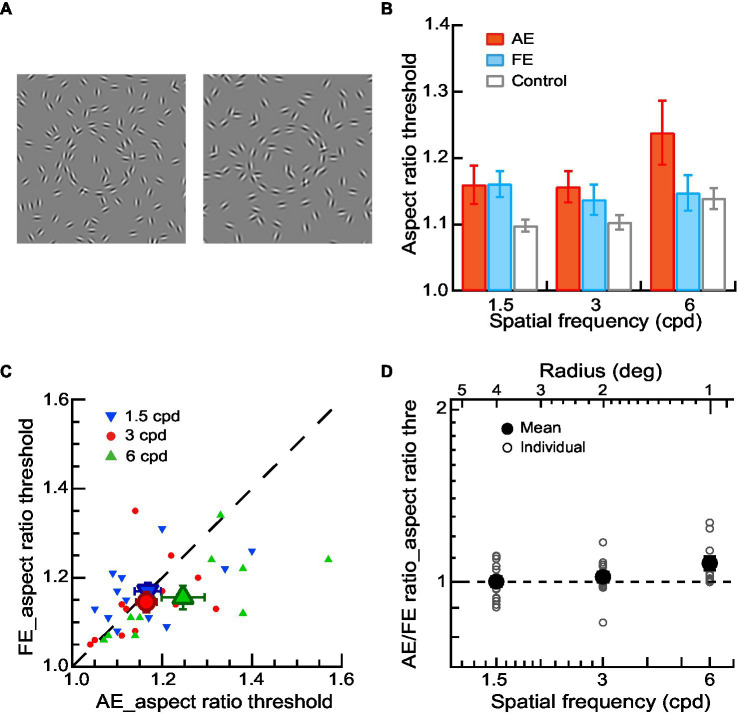
Shape perception of contours in amblyopic children and children with normal vision. **(A)** Circular or elliptical contours embedded in a noise background were used for a contour discrimination task. The aspect ratio thresholds were measured. **(B)** Mean aspect ratio thresholds of AEs (red bars), FEs (blue bars), and normal control eyes (gray bars) across different spatial frequencies. **(C)** Aspect ratio thresholds of FEs and AEs. Data points below the diagonal line indicate a trend of higher thresholds in AEs than in FEs. Gabor spatial frequency is coded by different symbols and colors. The larger dots show mean thresholds and the smaller ones show individuals’ data for each amblyopic observer. **(D)** The AE/FE ratio of aspect ratio threshold as a function of Gabor spatial frequency (lower abscissa). The radius (top abscissa) decreased proportionally as the Gabor spatial frequency increased. Data points above the dashed line indicate higher thresholds in the AEs than the FEs. The larger dots show mean thresholds and the smaller ones show individuals’ data for each amblyopic observer. Error bars indicate one standard error of the mean.

The stimuli were a full-screen field consisting of a contour of equally spaced Gabor elements in the shape of a circle or ellipse against a blank field (Figure 1A) or embedded in a field of noise Gabor patches (Figures 2A 3A). The center of the contour was positioned at the center of the stimulus. The screen was divided into 24 × 18 invisible square grids (432 in total), with a grid size of 1 deg. at a viewing distance of 1 meter. The noise Gabor was distributed in each grid with random orientations and positional jitter within ±0.5 grid size in both horizontal and vertical directions from the grid center. A contour Gabor element replaced a noise Gabor in the same grid to avoid density cues. The stimulus was regenerated in each interval. The spatial frequencies of Gabor elements were 1.5, 3, and 6 cpd at a viewing distance of 0.5, 1, and 2 meters, and the corresponding radii of the contour circle were 4, 2, and 1 degree. The standard deviation of the Gaussian envelope (σ) was 0.15°. The phases of neighboring contour Gabor patches alternated at 0 and 180 deg, while the phases of the noise Gabor patches were randomized at 0 or 180 deg. All contour and noise Gabor elements were physically identical except for their phases, locations, and orientations.

**Figure 3:**
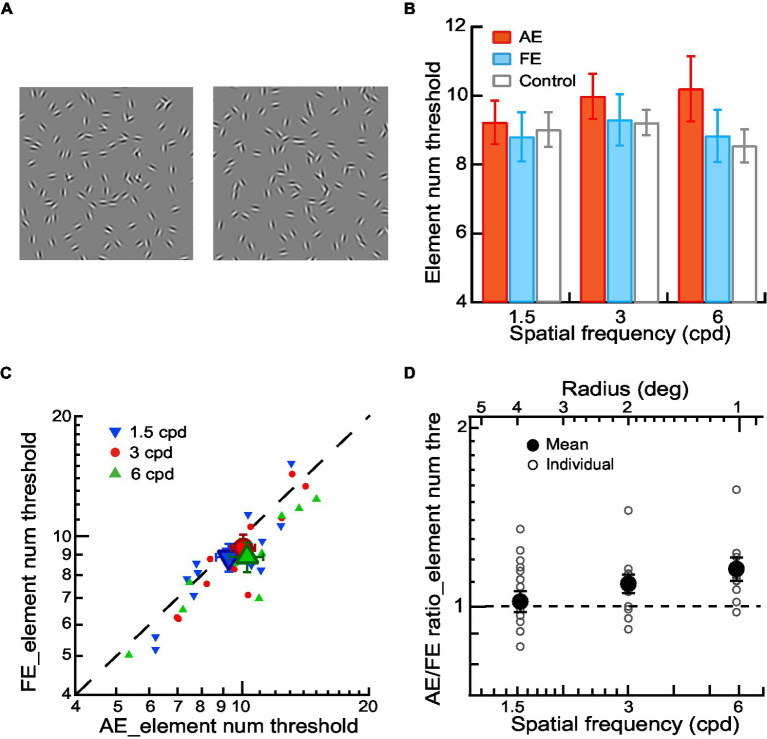
Contour integration in amblyopic children and children with normal vision. **(A)** Circular or elliptical contours embedded with a few elements in a noise background were used for contour discrimination experiments. The number of contour elements was measured as the thresholds of contour element number. **(B)** Mean element number thresholds of AEs (red bars), FEs (blue bars), and normal control eyes (gray bars) across different spatial frequencies. **(C)** Element number thresholds of FEs and AEs. Data points below the diagonal line indicate a trend of higher thresholds in AEs than in FEs. Gabor spatial frequency is coded by different symbols and colors. The larger dots show mean thresholds and the smaller ones show individuals’ data for each amblyopic observer. **(D)** The AE/FE ratio of element number threshold as a function of Gabor spatial frequency (lower abscissa). The radius (top abscissa) decreased proportionally as the Gabor spatial frequency increased. Data points above the dashed line indicate higher thresholds in the AEs than the FEs. The larger dots show mean thresholds and the smaller ones show individuals’ data for each amblyopic observer. Error bars indicate one standard error of the mean.

### Procedures

For amblyopic children, we measured contrast thresholds, aspect ratio thresholds, and contour element number thresholds for the AEs and FEs separately with a two-interval forced-choice (2IFC) staircase procedure in three experiments, respectively. In Experiment 1, the contrast detection thresholds were measured in a contour detection task. The two stimulus intervals were a circular contour made up of 10 Gabor patches (Figure 1A) and a blank field. The observers’ task was to judge which interval contained a contour. A staircase varied the contrast of Gabor trial by trial. In Experiment 2, the aspect ratio thresholds were measured in a contour discrimination task (Figure 2A). The number of contour elements was fixed at 15 and the aspect ratio of the elliptical contour was varied with a staircase to determine the aspect ratio thresholds. In Experiment 3, the thresholds of contour element numbers were measured with a staircase in a contour discrimination task (Figure 3A). The aspect ratio of the ellipse was held twice the aspect ratio threshold to minimize the effect of degraded shape perception of the AEs. In Experiments 2 and 3, the two stimulus intervals were circular and elliptical contours embedded in a field of the noise background. The observers’ task was to judge which interval contained an elliptical contour. The contrast of Gabor for the AEs was always equal to 90%, and that for the FEs was set to be an equal multiple of the contrast threshold to that of the AE. This was to make sure that the stimuli presented to the AEs and FEs had the same visibility. The contour was centered on the screen. The contour radius was fixed to 4, 2, and 1 degree, respectively, in different blocks to minimize the positional and shape uncertainty. For the elliptical contour, the axis of elongation was varied in each trial to make sure that the observers attend to the entire figure.

The two stimulus intervals were presented in a random order for 200 ms each, with an inter-stimulus interval of 500 ms. A fixation cross preceded the first stimulus interval by 500 ms. Auditory feedback was given on incorrect responses. A classical 3-down-1-up staircase rule that resulted in a 79.4% convergence level was used. Each staircase consisted of four preliminary reversals and six experimental reversals. A reversal occurs if the stimulus value moves up when it was last moved down, or vice versa. The step size of the staircase was 0.05 log units. The geometric mean of the experimental reversals was taken as the threshold for each staircase run. We varied the Gabor spatial frequency (1.5, 3, and 6 cpd) in separate runs by varying the observer’s viewing distance for each experiment, which also varied the radii of the contour circle. Each test condition was repeated 2 ~ 3 times and the thresholds reported are the geometric mean of the separate estimates. All observers participated in all experiments, except for two observers who missed several test conditions (Experiment 3: 6 cpd for S4, 3 cpd for S13).

The normal controls had the same procedure as the amblyopic group. In Experiments 2 and 3, the contrast of Gabor was set to be multiplied (the mean times of certain spatial frequency in FEs) by the contrast threshold of the normal control eyes. This was to make sure the stimuli presented to the FEs and normal control eyes had the same visibility.

### Visual function assessment

Visual acuities and contrast sensitivity functions for both eyes and the Randot stereoacuity were measured before contour integration testing.

#### Visual acuity

Visual acuity was assessed with the Chinese Tumbling E Chart (Mou, 1966), which has 14 lines, with the size of the optotypes ranging from 1 to 0.3 logMAR and changing by 0.1 log unit from line to line. Observers were required to report the orientation (the opening) of the letter E. Visual acuity is defined as the logMAR associated with 75% correct identification.

The visual crowding effect, which means visual acuity test results with a single optotype better than those with an array or a full chart of symbols, is marked in amblyopic eyes (Levi, 2008). To evaluate the crowding effects, we also measured single-E and crowded-E acuities with a custom computerized program at a viewing distance of 4 m as our previous studies used (Zhang et al., 2014; Liu and Zhang, 2018, 2019; Liu et al., 2021). Single-E acuity was measured with a tumbling letter E (a minimal luminance black letter on a full luminance white background). Crowded-E acuity was tested with a tumbling E letter target surrounded by four same-sized tumbling E flankers in the four cardinal directions, with an edge-to-edge gap of one letter size. The stroke and opening width of the E letter was one-fifth of the letter height. Note that S13 missed the data of single-E acuity and crowded-E acuity. The E acuities were all measured with a single-interval staircase procedure. The stimulus stayed on until a keypress by the observer. The task was to judge the orientation of the tumbling E (left, right, up, or down). All thresholds were estimated following a 3-down-1-up staircase rule. For efficient clinical testing, each staircase consisted of two preliminary reversals and four experimental reversals. The step size of the staircase was 0.05 log units. The geometric mean of the experimental reversals was taken as the threshold for each staircase run. Three staircases were run to determine single-E or crowded-E acuities.

#### Contrast sensitivity

Contrast sensitivity (i.e., the reciprocal of contrast threshold) for each eye was measured for amblyopic observers. The stimulus was a Gabor patch with a standard deviation of the Gaussian envelope (σ) of 0.9° and an orientation tilted ±45° from vertical. The spatial frequencies of the Gabor were 1, 3/4, 1/2, 1/4, and 1/16 times the cutoff spatial frequency, which was measured with a stimulus of a 0.29° × 0.29° sharp-edged full-contrast square-wave grating tilted ±45° from vertical. Both the contrast threshold and the cutoff frequency were established with a single-interval staircase procedure at a viewing distance of 4 meters. The observers were asked to judge the orientation of the stimulus (tilted to the left or right from vertical). Each staircase consisted of two preliminary reversals and six experimental reversals. The step size of the staircase was 0.05 log units for contrast threshold measurements and 0.03 log units for cutoff frequency measurements. For each measurement, three staircases were run consecutively for one eye before switching to the other. The order of all staircases followed a randomly permuted table, which was different for each observer’s AE and FE. The mean contrast sensitivity functions (CSFs) were fitted with a difference of Gaussians function: y=A1e−(xσ1)2−A2e−(xσ2)2 , where *y* is the contrast sensitivity, *x* is the spatial frequency, *A*_1_ and *A*_2_ are the amplitudes, and *σ*_1_ and *σ*_2_ are the standard deviations.

#### Stereoacuity

Stereoacuity was tested with the Randot Stereo Test (Stereo Optical Co, Inc., Chicago, IL) under normal room lighting. Contoured circles at 10 levels of disparity ranging from 400 to 20 arcsec provide a graded sequence for testing. Observers wore polarizing glasses and looked at the test material at a viewing distance of 40 cm. Note that the stereoacuity for those who failed the Randot Stereo Test was set at 500 arcsec, a value below the lowest measurable score, for the convenience of data analysis.

## Results

In three experiments, we measured the contrast thresholds, the aspect ratio thresholds, and the contour element number thresholds for the AEs and FEs at Gabor spatial frequencies of 1.5, 3, and 6 cpd in 13 anisometropic amblyopic children and investigated the contrast deficits, degraded shape perception, and contour integration deficits, respectively. A group of 13 children with normal vision participated as a control.

### Contrast detection of contour in amblyopic children and children with normal vision

In Experiment 1, a contour detection experiment was conducted to measure the contrast thresholds for the contour stimuli (circles comprised of 10 Gabor patches without noise, Figure 1A) at Gabor spatial frequencies of 1.5, 3, and 6 cpd. The average contrast thresholds of AEs, FEs, and normal control eyes were shown in Figure 1B. We conducted a two-way ANOVA with Gabor spatial frequency (1.5, 3, and 6 cpd) and eye (AEs vs. normal control eyes) as the repeated measures. The significant main effect on the eye [*F*(1,12) = 5.24, *p* = 0.04, η_p_^2^ = 0.30] was found, indicating a significant difference in contrast thresholds between AEs and normal control eyes (Figure 1B). We also conducted a two-way ANOVA to examine the difference in the contrast thresholds between FEs and normal control eyes with Gabor spatial frequency (1.5, 3, and 6 cpd) and eye (FEs vs. normal control eyes) as the repeated measures. No significant main effect was found on the eye (*p* = 0.58), which indicated no significant difference between the FEs and normal control eyes (Figure 1B).

Figure 1C shows the contrast detection thresholds of AEs and FEs for each amblyopic observer. To analyze the contrast deficits in amblyopic children between AEs and FEs, the contrast thresholds were entered into a two-way ANOVA with Gabor spatial frequency (1.5, 3, and 6 cpd) and eye (AEs vs. FEs) as the repeated measures. A significant difference in contrast thresholds between AEs and FEs [*F*(1,12) = 7.89, *p* = 0.02, η_p_^2^ = 0.40] was found, with higher contrast thresholds in the AEs (8.67% ± 3.53% at 1.5 cpd, 11.19% ± 5.15% at 3 cpd, 26.58% ± 20.24% at 6 cpd) than that in FEs (6.01% ± 1.95% at 1.5 cpd, 8.45% ± 3.21% at 3 cpd, 14.99% ± 5.21% at 6 cpd). A significant interaction effect between spatial frequency and the eye was also found [*F*(2,24) = 3.64, *p* = 0.04, η_p_^2^ = 0.23]. Pairwise comparisons indicated that this interaction was mainly due to the significant difference at different spatial frequencies between AEs and FEs (*p* = 0.01 at 1.5 cpd and *p* = 0.04 at 6 cpd).

To further analyze the contrast deficits in the AEs, we calculated the AE/FE ratio of contrast thresholds for each amblyopic observer (Figure 1D). A value of AE/FE ratio greater than one implies that the AE may have contrast deficits compared to the FE. We conducted one-sample *t*-tests at each spatial frequency (one-sample *t*-tests were used in the later analysis unless specified) and found that the average values of the AE/FE ratio were significantly greater than one at all spatial frequencies (*p* = 0.03 at 1.5 cpd, *p* = 0.03 at 3 cpd and *p* = 0.02 on 6 cpd), which indicates that there were contrast deficits in the AEs at both lower and higher spatial frequencies.

### Shape perception of contours in amblyopic children and children with normal vision

In Experiment 2, a contour discrimination task was performed to measure the aspect ratio thresholds of AEs, FEs, and normal control eyes. The observers were required to judge which interval contained an elliptical contour (Figure 2A). The number of contour elements was fixed at 15 and the aspect ratio of the elliptical contour was varied. The stimulus visibility was matched for AEs, FEs, and normal control eyes with the contrast thresholds measured in the first experiment (see Methods).

To examine shape perception deficits in the AEs, we conducted a two-way ANOVA with Gabor spatial frequency (1.5, 3, and 6 cpd) and eye (AEs vs. normal control eyes) as the repeated measures. The significant main effect on the eye [*F*(1,10) = 5.03, *p* = 0.04, η_p_^2^ = 0.34] was found, indicating a significant difference in the aspect ratio thresholds between AEs and normal control eyes. We also conducted a two-way ANOVA to examine the difference in the aspect ratio thresholds between FEs and normal control eyes with Gabor spatial frequency (1.5, 3, and 6 cpd) as a within-subject factor and eye (FEs vs. normal control eyes) as a between-subject factor. No significant main effect was found on the eye (*p* = 0.12), which indicated no significant difference between the FEs and normal control eyes (Figure 2B).

Figure 2C shows the aspect ratio thresholds of AEs and FEs for each amblyopic observer. The aspect ratio thresholds were entered into a two-way ANOVA with Gabor spatial frequency (1.5, 3, and 6 cpd) and eye (AEs vs. FEs) as the repeated measures. The significant interaction effect between spatial frequency and the eye was also found [*F*(2,20) = 5.27, *p* = 0.02, η_p_^2^ = 0.36] in aspect ratio thresholds. Pairwise comparisons indicated that this interaction was mainly due to the significant difference at 6 cpd between AEs (1.25 ± 0.16) and FEs (1.16 ± 0.09) (*p* = 0.03). These results suggest the AEs had degraded shape perception at the higher spatial frequencies (6 cpd) even when the contrast deficits were compensated for.

To further analyze the degraded shape perception in the AEs, we calculated the AE/FE ratio of aspect ratio thresholds for each amblyopic observer (Figure 2D). A value of AE/FE ratio greater than one implies that the AE may have degraded shape perception compared to the FE. We found that the average value of AE/FE ratio was significantly greater than one at the spatial frequency of 6 cpd (AE/FE ratio = 1.08 ± 0.10, *t_10_* = 2.68, *p* = 0.02, Cohen’s *d* = 0.81), but not at 1.5 cpd (AE/FE ratio = 1.00 ± 0.08, *t_12_* = −0.01, *p* = 0.99, Cohen’s *d* = −0.05) and 3 cpd (AE/FE ratio = 1.02 ± 0.08, *t_12_* = 0.94, *p* = 0.37, Cohen’s *d* = 0.26). These results suggest the AEs had degraded shape perception at higher spatial frequencies (e.g., 6 cpd) even when the contrast deficits were compensated for. Note that two observers (S5 and S12) were exempted from the two contour discrimination tasks at the spatial frequency of 6 cpd due to their remarkably high contrast thresholds in the AEs. This was to make sure that the contrast of the stimuli used in the contour discrimination tasks for the AEs was at least 2.5 times the contrast thresholds measured in the contour detection task.

### Contour integration in amblyopic children and children with normal vision

In Experiment 3, we measured the element number thresholds of AEs, FEs, and normal control eyes in a contour discrimination task while compensating for the low-level deficits of decreased contrast sensitivity and degraded shape perception in the AEs (Figure 3A). The stimulus visibility was equalized for AEs and FEs, and the aspect ratio of the elliptical contour was set to twice the aspect ratio threshold to eliminate the influence of abnormal shape perception of the AEs (see Methods).

To investigate contour integration deficits in the AEs, We conducted a two-way ANOVA with Gabor spatial frequency (1.5, 3, and 6 cpd) and eye (AEs vs. normal control eyes) the repeated measures. The significant main effect on the eye [*F*(1,8) = 7.76, *p* = 0.02, η_p_^2^ = 0.49] was found, indicating a significant difference in the element number thresholds between AEs and normal control eyes. We also conducted a two-way ANOVA to examine the difference in the element number thresholds between FEs and normal control eyes with Gabor spatial frequency (1.5, 3, and 6 cpd) and eye (FEs vs. normal control eyes) as the repeated measures. No significant main effect was found in the eye (*p* = 0.23), which indicated no significant difference between the FEs and normal control eyes (Figure 3B).

Figure 3C shows the element number thresholds of AEs and FEs. The element number thresholds were entered into a two-way ANOVA with Gabor spatial frequency (1.5, 3, and 6 cpd) and eye (AEs vs. FEs) as the repeated measures. We found a significant main effect on the eye [*F*(1,8) = 6.25, *p* = 0.04, η_p_^2^ = 0.44] and a significant interaction effect between spatial frequency and the eye [*F*(1,16) = 4.32, *p* = 0.03, η_p_^2^ = 0.35]. Pairwise comparisons indicated that this interaction was mainly due to the significant difference at different spatial frequencies between AEs and FEs (*p* = 0.04 at 3 cpd and *p* = 0.01 at 6 cpd).

To further analyze the contour integration deficits in the AEs, we calculated the AE/FE ratio of element number thresholds for each amblyopic observer to see the deficits after matching the contrast threshold and the aspect ratio thresholds (Figure 3D). A value of the AE/FE ratio greater than one implies that the AE may have contour integration deficits compared to the FE. We found that the average values of AE/FE ratio were significantly greater than one at the spatial frequency of 3 cpd (AE/FE ratio = 1.09 ± 0.14, *t_11_* = 2.33, *p* = 0.04, Cohen’s *d* = 0.67) and 6 cpd (AE/FE ratio = 1.15 ± 0.17, *t_9_* = 2.99, *p* = 0.02, Cohen’s *d* = 0.95), but not at 1.5 cpd (AE/FE ratio = 1.07 ± 0.15, *t_12_* = 1.58, *p* = 0.14, Cohen’s *d* = 0.44). These results showed that there existed impairments in contour integration at higher Gabor spatial frequencies (3 and 6 cpd) in amblyopic children, even after the low-level deficits of degraded visibility and abnormal shape perception in the AEs were compensated for.

### Contrast sensitivity functions in amblyopic children

**Figure 4:**
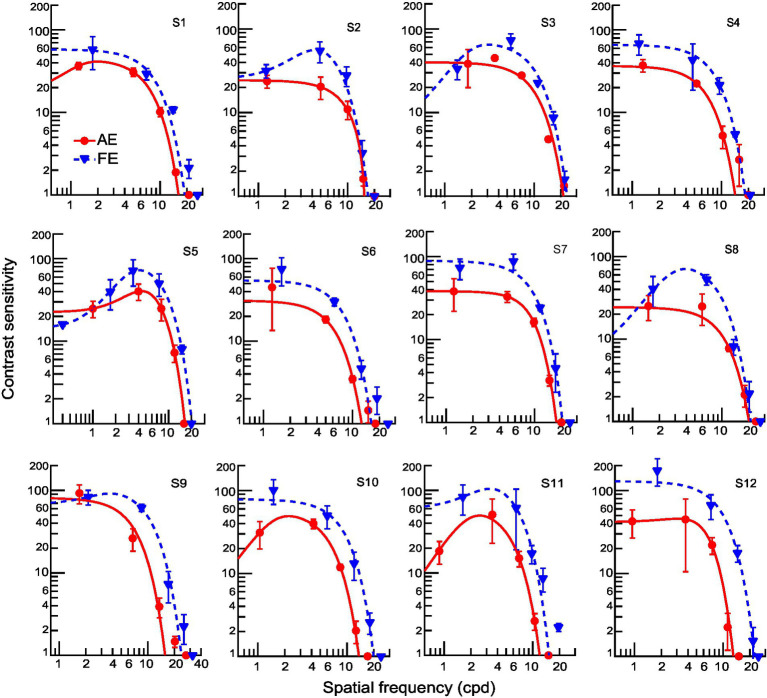
Individual contrast sensitivity functions for AEs (red) and FEs (blue). Each curve is the best fitting of a Difference-of-Gaussian function (AE: solid line; FE: dashed line). One observer (S13) did not complete the contrast sensitivity assessment.

For amblyopic children, individuals’ contrast sensitivity functions (CSFs) fitted with a difference of Gaussians function were plotted in Figure 4. Because the loss of contrast sensitivity in amblyopes varies with spatial frequency, the severity of amblyopia cannot be captured by observing a single point on the curve, such as the peak or the cutoff frequency. A more inclusive measurement is required to account for the spatial frequencies to which the observer is sensitive. Therefore, we calculated the area under the log CSF (AULCSF) to estimate the overall contrast sensitivity for each amblyopic observer’s AE and FE. The AULCSF for the AE was then divided by the AULCSF of the FE to determine the interocular differences in AULCSF for each observer (abbreviated as ‘AE/FE ratio_AULCSF’). The values of AE/FE ratio_AULCSF were significantly less than one (one sample *t*-test, *t_11_* =  −14.23, *p* < 0.001, Cohen’s *d* = 4.11), indicating that the AEs have losses in contrast sensitivity. In addition, the values of AE/FE ratio_AULCSF were significantly correlated to the interocular acuity difference in amblyopic children (*r* = 0.62, *p* = 0.003), suggesting that both indices may be useful in characterizing the severity of amblyopia. We also found that the AE/FE ratio_AULCSF and the interocular acuity difference significantly correlated with the AE/FE ratio of contrast thresholds at 6 cpd (*r* = −0.59, *p* = 0.042, and *r* = 0.62, *p* = 0.003, respectively), but not at 1.5 cpd and 3 cpd (all *p*s > 0.05), demonstrating that AEs showed a loss of contrast sensitivity and spatial resolution at higher spatial frequencies.

### Relationship between visual functions and contour integration deficits in amblyopic children

**Figure 5:**
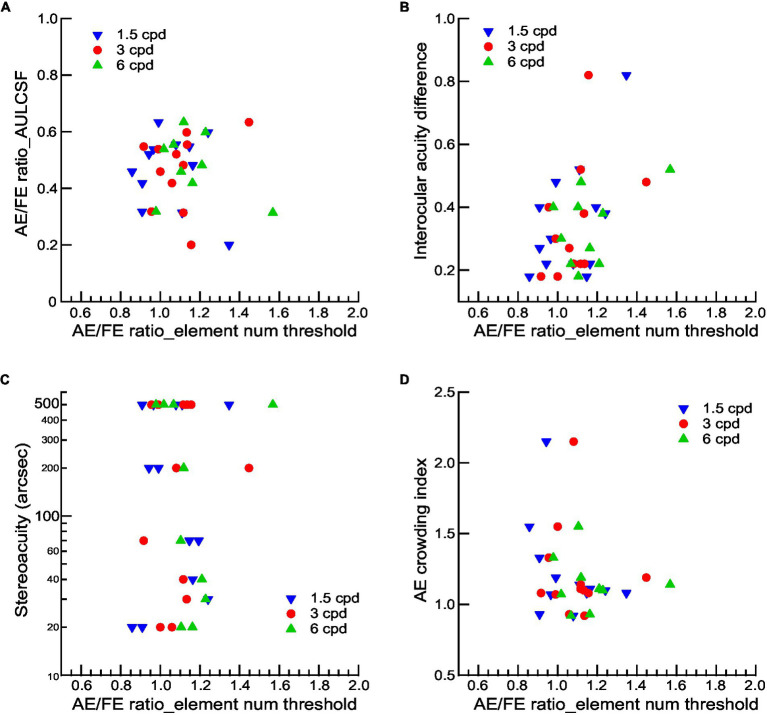
The relationship between visual functions and contour integration deficits for amblyopic observers. AE/FE ratio_AULCSF **(A)**, interocular acuity difference **(B)**, stereoacuity **(C)**, and crowding index of AEs **(D)** as a function of the AE/FE ratio of element number thresholds at three Gabor spatial frequencies. Gabor spatial frequency is coded by different symbols and colors. Note that the stereoacuity for those who failed the Randot Stereo Test was set at 500 arcsec, a value below the lowest measurable score, for the convenience of data analysis.

To assess the potential affecting factor of visual functions to contour integration deficits in amblyopic children, we used Pearson’s r correlation and found that the AE/FE ratio_AULCSF and the interocular acuity difference have no significant correlation with the AE/FE ratio of the element number threshold at any spatial frequency (Figures 5A,B, all *p*s > 0.05), suggesting that the contour integration deficits were not related to basic losses in contrast sensitivity and visual acuity after eliminating or compensating for the low-level deficits (reduced visibility, increased positional uncertainty, and abnormal shape perception). We also examined the associations between binocularity (as indexed by stereopsis) and contour integration deficits in amblyopic children and found no significant correlations between the stereoacuity and the AE/FE ratio of the element number threshold at any spatial frequency (Figure 5C, all *p*s > 0.05), suggesting that contour integration deficits were not associated with binocularity in amblyopic children.

Since the viewing distance was changed, the size of the contour and the spacing of the contour elements were varied by different spatial frequencies, which may lead to potential crowding effects. We quantified crowding effects with a crowding index, which was defined as the ratio of crowded-E acuities to single-E acuities (see Methods). The crowding index was significantly greater than 1 (Mean ± SD = 1.21 ± 0.06, *t_11_* = 3.619, *p* = 0.004, Cohen’s *d* = 4.11), indicating the existence of visual crowding in AEs. However, we found no significant correlations between the crowding index and the AE/FE ratio of the element number threshold at any spatial frequency (Figure 5D, all *p*s > 0.05), suggesting that contour integration deficits at higher Gabor spatial frequencies might not be attributed to the effects of visual crowding.

All amblyopic children had received refractive correction and/or occlusion therapy before the testing, although the treatment length is variable in different observers. The results showed that there was no correlation of treatment length to the AE/FE ratio of the element number threshold at any spatial frequency (all *p*s > 0.05).

## Discussion

The current study investigated contour integration deficits in children treated for anisometropic amblyopia. We found that the AEs showed reduced contrast sensitivity and degraded shape perception at higher spatial frequencies (e.g., 6 cpd). Even after compensating for reduced contrast sensitivity and decreased shape perception in the AEs, we still found contour integration deficits in the AEs compared to the FEs at spatial frequencies 3 and 6 cpd. These deficits were not related to basic losses in contrast sensitivity and acuity, stereoacuity, and visual crowding. Besides, no significant difference was found between the fellow eyes of the amblyopic children and the normal control eyes in the performance of contour integration.

The current study provides further evidence for contour integration deficits in the AEs compared to FEs, which is consistent with previous findings (Chandna et al., 2001, 2004). Here we used rigorous psychophysical methods that require global contour processing while minimizing the influence of low-level deficits (visibility, shape perception, and positional uncertainty), and we found contour integration deficits mainly present at higher frequencies (3 and 6 cpd), which is in line with the idea that most of the deficiency in the AEs occurs at mid to high spatial frequencies (Levi, 2020; Chen et al., 2021). The residual deficits found in the contour integration experiments were more likely to be impairments in contour processing (Hamm et al., 2014). These findings are consistent with the studies using other higher-order spatial stimuli such as glass patterns, which also found more impairments at higher spatial frequencies in amblyopia (Rislove et al., 2005, 2010). These results indicated deficits in processing global form at the finest spatial scale in amblyopia. Besides, our results showed that AEs have significant deficits in contrast detection, shape perception, and contour integration compared to the normal control eyes, which provides additional evidence for contour integration deficits in children treated for anisometropic amblyopia. However, we did not find visual function impairments in the fellow eyes (FEs) compared to normal control eyes (Meier and Giaschi, 2017; Birch et al., 2019), as the normal control eyes showed no significant difference between the fellow eyes of the amblyopic children in the performance of contrast detection, shape perception, and contour integration.

We found that the severity of amblyopia was correlated to contour contrast detection, as the AE/FE ratio_AULCSF and the interocular acuity difference significantly correlated with the AE/FE ratio of contrast thresholds at 6 cpd. These results are consistent with previous studies that reported correlations between interocular differences in contour integration and contrast sensitivity (Benedek et al., 2010). However, we did not find significant correlations between contour integration deficits and the severity of amblyopia (Figures 5A,B), consistent with some previous studies that showed insignificant correlations between contour integration deficits with the basic losses in visual acuity and contrast sensitivity either (Kozma and Kiorpes, 2003; Levi et al., 2007). For example, Kozma and Kiorpes (2003) reported that the contour integration deficits were not related to acuity and contrast sensitivity for either anisometropic or strabismic amblyopic monkeys. Moreover, contour integration deficits did not relate to the stereoacuity nor to the visual crowding, which was consistent with the results of amblyopic adults (Levi et al., 2007). These findings suggest that the abnormal contour integration in amblyopic children might reflect the impairments of contour processing *per se*. Therefore, further research in amblyopia needs thorough examinations of visual functions, especially those processed at the mid and high levels, and effective treatment and training methods should be developed to recover these deficits.

Chandna et al. (2004) found that contour integration deficits in amblyopic children could fully recover with several weeks of refractive correction alone or in combination with occlusion therapy, as they found that interocular differences in contour detection thresholds declined to normal levels in most of the patients within 8 weeks of the initiation of treatment. In contrast, in our group of amblyopic children, all of whom had received refractive correction and/or occlusion therapy with different ranges of treatment length (ranging from 2 to 73.1 months), we still found contour integration deficits, mainly at higher spatial frequencies. Indeed, there are some differences in the tasks and stimuli between ours and the study of Chandna et al. (2004). For example, we used a computer-based contour discrimination task and varied the number of contour elements while keeping the density of background noise constant, whereas Chandna et al. (2004) measured the contrast threshold with a contour detection task presented with cards and varied the density of background noise while keeping the contour element spacing constant. The stimuli we used were perfect circular and ellipse contours centered on the screen to minimize the positional and shape uncertainty, while those in Chandna et al. (2004) were nearly circular contours located near one of the four corners. These differences in the tasks and stimuli might result in different measuring sensitivity of contour integration (Mathes and Fahle, 2007).

On the other hand, there might exist some age-related changes in the ability of contour integration. We noticed that the mean age of the amblyopic children in Chandna et al. (2004) (mean = 7.0 years) is smaller than ours (mean = 10.1 years). Though there is evidence showing a gradual improvement in contour integration throughout childhood in normal vision and the slow development of sensitivity to the statistics of natural scenes (Hadad et al., 2010), we still found contour integration deficits in older amblyopic children. We also noticed that the age at which treatment was performed differed between children in Chandana’s study (mean = 7.0 years, all were children with previously untreated amblyopia) and children in our study (mean ± SD = 7.5 ± 2.9 years). The consequently reduced plasticity of contour integration is also a potential factor if the plasticity gradually decreases with age, this is indeed the case for many visual functions (Park and Fine, 2020; Mitchell and Maurer, 2022). Given the heterogeneous nature of amblyopia, these related factors might lead to differences between studies. Nevertheless, whether conventional treatments are sufficient to completely restore contour integration deficits in amblyopic children needs further investigation.

The contour integration deficits in amblyopia might be related to deficits in not only the striate cortex but also the higher visual areas. Neurophysiological studies in monkeys have identified that V1 is intimately involved in contour integration (Polat et al., 1998; Kapadia et al., 2000; Li and Gilbert, 2002; Li et al., 2006). On the other hand, neuroimaging studies have identified that both striate and extrastriate areas contribute to contour integration (Altmann et al., 2003; Kourtzi et al., 2003; Kuai et al., 2017). Moreover, accumulating evidence suggests that contour processing is strongly dependent on top-down feedback influences (Chen et al., 2014; Mijovic et al., 2014; Li et al., 2019). Our results suggested the abnormal contour integration in amblyopes might reflect that the disruption of mechanisms is different from those that determine acuity and contrast sensitivity (Kozma and Kiorpes, 2003). Besides, there is evidence showing that the maturation of contour detection mechanisms depends at least in part on the presence of normal binocular interaction during a developmental critical period (Norcia et al., 2005). Given that the underlying neural mechanisms of both contour integration and the neural deficits of amblyopia involve multiple visual areas from lower to higher levels (Kiorpes and Daw, 2018), our findings suggest that contour processing deficits in amblyopia may involve impairments not only in the early but also in the high-level visual cortex.

Our current study has its limitation. First, our results are based on anisometropic amblyopic children. There is evidence showing that anisometropic amblyopia has less severe consequences for contour integration mechanisms than strabismic amblyopia, which produces a different pattern of loss and waveform abnormalities (Norcia et al., 2005). Therefore, further evidence from strabismic amblyopes is necessary for a more balanced evaluation of contour integration and global processing in amblyopic children. Second, our findings may be specific to the task used in the current study, and the underlying mechanisms of this task are not well understood. Different tests on contour integration were used in adults with amblyopia (Hess et al., 1997; Hess and Demanins, 1998; Kovács et al., 2000; Levi et al., 2007). For the anisometropic amblyopic adults, while Hess and Demanins (1998) found no contour integration deficits in most observers with a contour detection task, Levi et al. (2007) found genuine contour integration deficits in two of three observers with the same tasks in the current study. Further investigation into the mechanisms underlying different contour integration tasks and evaluation of the effectiveness of different tasks are needed.

## Data availability statement

The raw data supporting the conclusions of this article will be made available by the authors, without undue reservation.

## Ethics statement

The studies involving human participants were reviewed and approved by The ethics committees of Tengzhou Central People’s Hospital and Peking University. Written informed consent to participate in this study was provided by the participants’ legal guardian/next of kin.

## Author contributions

J-YZ conceived and designed the study. S-QJ, Y-RC, and X-YL performed the experiments. S-QJ, Y-RC, and J-YZ analyzed the data and wrote the manuscript. All authors read and approved the manuscript.

## Funding

This research was supported by the Natural Science Foundation of China grants 31970978 (J-YZ; Beijing, China).

## Conflict of interest

The authors declare that the research was conducted in the absence of any commercial or financial relationships that could be construed as a potential conflict of interest.

## Publisher’s note

All claims expressed in this article are solely those of the authors and do not necessarily represent those of their affiliated organizations, or those of the publisher, the editors and the reviewers. Any product that may be evaluated in this article, or claim that may be made by its manufacturer, is not guaranteed or endorsed by the publisher.

## References

[ref1] Altmann C. F. Bülthoff H. H. Kourtzi Z. (2003). Perceptual organization of local elements into global shapes in the human visual cortex. Curr. Biol. 13, 342–349. doi: 10.1016/S0960-9822(03)00052-6 12593802

[ref2] Awan M. Proudlock F. A. Gottlob I. (2005). A randomized controlled trial of unilateral strabismic and mixed amblyopia using occlusion dose monitors to record compliance. Invest. Ophthalmol. Vis. Sci. 46, 1435–1439. doi: 10.1167/iovs.04-0971 15790912

[ref3] Barnes G. R. Hess R. F. Dumoulin S. O. Achtman R. L. Pike G. B. (2001). The cortical deficit in humans with strabismic amblyopia. J. Physiol. 533, 281–297. doi: 10.1111/j.1469-7793.2001.0281b.x 11351035 PMC2278601

[ref4] Bauer R. Heinze S. (2002). Contour integration in striate cortex - classic cell responses or cooperative selection? Exp. Brain Res. 147, 145–152. doi: 10.1007/s00221-002-1178-6 12410329

[ref5] Benedek K. Janáky M. Braunitzer G. Rokszin A. Kéri S. Benedek G. (2010). Parallel development of contour integration and visual contrast sensitivity at low spatial frequencies. Neurosci. Lett. 472, 175–178. doi: 10.1016/j.neulet.2010.02.001 20138967

[ref6] Bi H. Zhang B. Tao X. Harwerth R. S. Smith E. L. 3rd Chino Y. M. (2011). Neuronal responses in visual area V2 (V2) of macaque monkeys with strabismic amblyopia. Cereb. Cortex 21, 2033–2045. doi: 10.1093/cercor/bhq272 21263036 PMC3155601

[ref7] Birch E. E. (2013). Amblyopia and binocular vision. Prog. Retin. Eye Res. 33, 67–84. doi: 10.1016/j.preteyeres.2012.11.001 23201436 PMC3577063

[ref8] Birch E. E. Kelly K. R. Giaschi D. E. (2019). Fellow eye deficits in amblyopia. J Binocul Vis Ocul Motil 69, 116–125. doi: 10.1080/2576117X.2019.1624440 31161888 PMC6673659

[ref9] Birch E. E. Swanson W. H. (2000). Hyperacuity deficits in anisometropic and strabismic amblyopes with known ages of onset. Vis. Res. 40, 1035–1040. doi: 10.1016/s0042-6989(00)00011-0 10738062

[ref10] Bradley A. Freeman R. D. (1981). Contrast sensitivity in anisometropic amblyopia. Invest. Ophthalmol. Vis. Sci. 21, 467–476. 7275532

[ref11] Chandna A. Gonzalez-Martin J. A. Norcia A. M. (2004). Recovery of contour integration in relation to LogMAR visual acuity during treatment of amblyopia in children. Invest. Ophthalmol. Vis. Sci. 45, 4016–4022. doi: 10.1167/iovs.03-0795 15505051

[ref12] Chandna A. Pennefather P. M. Kovács I. Norcia A. M. (2001). Contour integration deficits in anisometropic amblyopia. Invest. Ophthalmol. Vis. Sci. 42, 875–878. 11222553

[ref13] Chen S. J. Min S. H. Cheng Z. Y. Xiong Y. Yu X. Wei L. L.. (2021). Binocular visual deficits at mid to high spatial frequency in treated amblyopes. Iscience 24. doi: 10.1016/j.isci.2021.102727 PMC8254032 34258558

[ref14] Chen M. Yan Y. Gong X. Gilbert C. D. Liang H. Li W. (2014). Incremental integration of global contours through interplay between visual cortical areas. Neuron 82, 682–694. doi: 10.1016/j.neuron.2014.03.023 24811385

[ref15] Clarke M. P. Wright C. M. Hrisos S. Anderson J. D. Henderson J. Richardson S. R. (2003). Randomised controlled trial of treatment of unilateral visual impairment detected at preschool vision screening. BMJ 327:1251. doi: 10.1136/bmj.327.7426.1251 14644966 PMC286242

[ref16] Conner I. P. Odom J. V. Schwartz T. L. Mendola J. D. (2007). Monocular activation of V1 and V2 in amblyopic adults measured with functional magnetic resonance imaging. J. AAPOS 11, 341–350. doi: 10.1016/j.jaapos.2007.01.119 17434776 PMC2174609

[ref17] Dallala R. Wang Y. Z. Hess R. F. (2010). The global shape detection deficit in strabismic amblyopia: contribution of local orientation and position. Vis. Res. 50, 1612–1617. doi: 10.1016/j.visres.2010.05.023 20510268

[ref18] Demanins R. Hess R. F. (1996). Positional loss in strabismic amblyopia: inter-relationship of alignment threshold, bias, spatial scale and eccentricity. Investig. Ophthalmol. Vis. Sci. 36, 2771–2794. doi: 10.1016/0042-6989(95)00318-5 8917764

[ref19] El-Shamayleh Y. Kiorpes L. Kohn A. Movshon J. A. (2010). Visual motion processing by neurons in area MT of macaque monkeys with experimental amblyopia. J. Neurosci. 30, 12198–12209. doi: 10.1523/JNEUROSCI.3055-10.2010 20826682 PMC2953773

[ref20] Field D. J. Hayes A. Hess R. F. (1993). Contour integration by the human visual system: evidence for a local "association field". Vis. Res. 33, 173–193. doi: 10.1016/0042-6989(93)90156-q 8447091

[ref21] Giaschi D. Lo R. Narasimhan S. Lyons C. Wilcox L. M. (2013). Sparing of coarse stereopsis in stereodeficient children with a history of amblyopia. J Vision 13:17. doi: 10.1167/13.10.17 23986537

[ref22] Gilad A. Meirovithz E. Slovin H. (2013). Population responses to contour integration: early encoding of discrete elements and late perceptual grouping. Neuron 78, 389–402. doi: 10.1016/j.neuron.2013.02.013 23622069

[ref23] Hadad B. Maurer D. Lewis T. L. (2010). The effects of spatial proximity and collinearity on contour integration in adults and children. Vis. Res. 50, 772–778. doi: 10.1016/j.visres.2010.01.021 20149911

[ref24] Hamm L. M. Black J. Dai S. Thompson B. (2014). Global processing in amblyopia: a review. Front. Psychol. 5:583. doi: 10.3389/fpsyg.2014.00583 24987383 PMC4060804

[ref25] Hess R. F. Demanins R. (1998). Contour integration in anisometropic amblyopia. Vis. Res. 28, 889–894. doi: 10.1016/s0042-6989(97)00233-2 9624438

[ref26] Hess R. F. Holliday I. E. (1992). The spatial localization deficit in amblyopia. Vis. Res. 32, 1319–1339. doi: 10.1016/0042-6989(92)90225-8 1455705

[ref27] Hess R. F. Howell E. R. (1977). The threshold contrast sensitivity function in strabismic amblyopia: evidence for a two type classification. Vis. Res. 17, 1049–1055. doi: 10.1016/0042-6989(77)90009-8 595414

[ref28] Hess R. F. McIlhagga W. Field D. J. (1997). Contour integration in strabismic amblyopia: the sufficiency of an explanation based on positional uncertainty. Vis. Res. 37, 3145–3161. doi: 10.1016/s0042-6989(96)00281-7 9463696

[ref29] Hess R. F. Wang Y. Z. Demanins R. Wilkinson F. Wilson H. R. (1999). A deficit in strabismic amblyopia for global shape detection. Vis. Res. 39, 901–914. doi: 10.1016/s0042-6989(98)00157-6 10341944

[ref30] Holmes J. M. Clarke M. P. (2006). Amblyopia. Lancet 367, 1343–1351. doi: 10.1016/s0140-6736(06)68581-4 16631913

[ref31] Holmes J. M. Kraker R. T. Beck R. W. Birch E. E. Cotter S. A. Everett D. F.. (2003). A randomized trial of prescribed patching regimens for treatment of severe amblyopia in children. Ophthalmology 110, 2075–2087. doi: 10.1016/j.ophtha.2003.08.001 14597512

[ref32] Kapadia M. K. Ito M. Gilbert C. D. Westheimer G. (1995). Improvement in visual sensitivity by changes in local context – parallel studies in human observers and in V1 of alert monkeys. Neuron 15, 843–856. doi: 10.1016/0896-6273(95)90175-2 7576633

[ref33] Kapadia M. K. Westheimer G. Gilbert C. D. (2000). Spatial distribution of contextual interactions in primary visual cortex and in visual perception. J. Neurophysiol. 84, 2048–2062. doi: 10.1152/jn.2000.84.4.2048 11024097

[ref34] Kiorpes L. Daw N. (2018). Cortical correlates of amblyopia. Vis. Neurosci. 35:E016. doi: 10.1017/S0952523817000232 29905122

[ref35] Kiorpes L. Kiper D. C. O'Keefe L. P. Cavanaugh J. R. Movshon J. A. (1998). Neuronal correlates of amblyopia in the visual cortex of macaque monkeys with experimental strabismus and anisometropia. J. Neurosci. 18, 6411–6424. doi: 10.1523/JNEUROSCI.18-16-06411.1998 9698332 PMC6793177

[ref36] Kourtzi Z. Tolias A. S. Altmann C. F. Augath M. Logothetis N. K. (2003). Integration of local features into global shapes: monkey and human fMRI studies. Neuron 23, 333–346. doi: 10.1016/s0896-6273(02)01174-1 12546827

[ref37] Kovacs I. Kozma P. Feher A. Benedek G. (1999). Late maturation of visual spatial integration in humans. Proc. Natl. Acad. Sci. U. S. A. 96, 12204–12209. doi: 10.1073/pnas.96.21.12204 10518600 PMC18436

[ref38] Kovács I. Polat U. Pennefather P. M. Chandna A. Norcia A. M. (2000). A new test of contour integration deficits in patients with a history of disrupted binocular experience during visual development. Vis. Res. 40, 1775–1783. doi: 10.1016/s0042-6989(00)00008-0 10814762

[ref39] Kozma P. Kiorpes L. (2003). Contour integration in amblyopic monkeys. Vis. Neurosci. 20, 577–588. doi: 10.1017/s0952523803205113 14977336

[ref40] Kuai S. G. Li W. Yu C. Kourtzi Z. (2017). Contour integration over time: psychophysical and fMRI evidence. Cereb. Cortex 27, 3042–3051. doi: 10.1093/cercor/bhw147 27242029

[ref41] Lee S. Y. Isenberg S. J. (2003). The relationship between stereopsis and visual acuity after occlusion therapy for amblyopia. Ophthalmology 110, 2088–2092. doi: 10.1016/s0161-6420(03)00865-0 14597513

[ref42] Levi D. M. (2008). Crowding--an essential bottleneck for object recognition: a mini-review. Vis. Res. 48, 635–654. doi: 10.1016/j.visres.2007.12.009 18226828 PMC2268888

[ref43] Levi D. M. (2013). Linking assumptions in amblyopia. Vis. Neurosci. 30, 277–287. doi: 10.1017/S0952523813000023 23879956 PMC5533593

[ref44] Levi D. M. (2020). Rethinking amblyopia 2020. Vis. Res. 176, 118–129. doi: 10.1016/j.visres.2020.07.014 32866759 PMC7487000

[ref45] Levi D. M. Harwerth R. S. (1977). Spatio-temporal interactions in anisometropic and strabismic amblyopia. Invest. Ophthalmol. Vis. Sci. 16, 90–95. 832970

[ref46] Levi D. M. Klein S. A. (1986). Sampling in spatial vision. Nature 320, 360–362. doi: 10.1038/320360a0 3960118

[ref47] Levi D. M. McKee S. P. Movshon J. A. (2011). Visual deficits in anisometropia. Vis. Res. 51, 48–57. doi: 10.1016/j.visres.2010.09.029 20932989 PMC3010510

[ref48] Levi D. M. Yu C. Kuai S. G. Rislove E. (2007). Global contour processing in amblyopia. Vis. Res. 47, 512–524. doi: 10.1016/j.visres.2006.10.014 17223155 PMC1851910

[ref49] Li W. Gilbert C. D. (2002). Global contour saliency and local colinear interactions. J. Neurophysiol. 88, 2846–2856. doi: 10.1152/jn.00289.2002 12424317

[ref50] Li W. Piech V. Gilbert C. D. (2006). Contour saliency in primary visual cortex. Neuron 50, 951–962. doi: 10.1016/j.neuron.2006.04.035 16772175

[ref51] Li Y. Wang Y. Li S. (2019). Recurrent processing of contour integration in the human visual cortex as revealed by fMRI-guided TMS. Cereb. Cortex 29, 17–26. doi: 10.1093/cercor/bhx296 29161359

[ref52] Liang H. L. Gong X. J. Chen M. G. Yan Y. Li W. Gilbert C. D. (2017). Interactions between feedback and lateral connections in the primary visual cortex. Proc. Natl. Acad. Sci. U. S. A. 114, 8637–8642. doi: 10.1073/pnas.1706183114 28739915 PMC5559040

[ref53] Liu X. Y. Zhang J. Y. (2018). Dichoptic training in adults with amblyopia: additional stereoacuity gains over monocular training. Vis. Res. 152, 84–90. doi: 10.1016/j.visres.2017.07.002 28736224

[ref54] Liu X. Y. Zhang J. Y. (2019). Dichoptic De-masking learning in adults with amblyopia and its mechanisms. Invest. Ophthalmol. Vis. Sci. 60, 2968–2977. doi: 10.1167/iovs.18-26483 31307059

[ref55] Liu X. Y. Zhang Y. W. Gao F. Chen F. Zhang J. Y. (2021). Dichoptic perceptual training in children with amblyopia with or without patching history. Invest. Ophthalmol. Vis. Sci. 62:4. doi: 10.1167/iovs.62.6.4 PMC8107508 33944893

[ref56] Mathes B. Fahle M. (2007). Closure facilitates contour integration. Vis. Res. 47, 818–827. doi: 10.1016/j.visres.2006.11.014 17286999

[ref57] McKee S. P. Levi D. M. Movshon J. A. (2003). The pattern of visual deficits in amblyopia. J Vision 3, 380–405. doi: 10.1167/3.5.5 12875634

[ref58] Meier K. Giaschi D. (2017). Unilateral amblyopia affects two eyes: fellow eye deficits in amblyopia. Invest. Ophthalmol. Vis. Sci. 58, 1779–1800. doi: 10.1167/iovs.16-20964 28346616

[ref59] Mijovic B. De Vos M. Vanderperren K. Machilsen B. Sunaert S. Van Huffel S.. (2014). The dynamics of contour integration: a simultaneous EEG-fMRI study. NeuroImage 88, 10–21. doi: 10.1016/j.neuroimage.2013.11.032 24269572

[ref60] Mitchell D. E. Maurer D. (2022). Critical periods in vision revisited. Annu. Rev. Vis. Sci. 8, 291–321. doi: 10.1146/annurev-vision-090721-110411 35385674

[ref61] Moseley M. J. Neufeld M. McCarry B. Charnock A. McNamara R. Rice T.. (2002). Remediation of refractive amblyopia by optical correction alone. Ophthalmic Physiol. Opt. 22, 296–299. doi: 10.1046/j.1475-1313.2002.00034.x 12162480

[ref62] Mou T. (1966). Logarithmic visual acuity chart and five-score recording. Chin. J. Ophthalmol. 13, 96–106.

[ref63] Mussap A. J. Levi D. M. (2000). Amblyopic deficits in detecting a dotted line in noise. Vis. Res. 40, 3297–3307. doi: 10.1016/s0042-6989(00)00154-1 11008145

[ref64] Norcia A. M. Sampath V. Hou C. Pettet M. W. (2005). Experience-expectant development of contour integration mechanisms in human visual cortex. J. Vis. 5, 116–130. doi: 10.1167/5.2.3 15831072

[ref65] Park W. J. Fine I. (2020). New insights into cortical development and plasticity: from molecules to behavior. Curr. Opin. Physio. 16, 50–60. doi: 10.1016/j.cophys.2020.06.004 PMC7480792 32923755

[ref66] Pelli D. G. (1997). The VideoToolbox software for visual psychophysics: transforming numbers into movies. Spat. Vis. 10, 437–442. 9176953

[ref67] Polat U. Mizobe K. Pettet M. W. Kasamatsu T. Norcia A. M. (1998). Collinear stimuli regulate visual responses depending on cell's contrast threshold. Nature 391, 580–584. doi: 10.1038/35372 9468134

[ref68] Repka M. X. Beck R. W. Holmes J. M. Birch E. E. Chandler D. L. Cotter S. A.. (2003). A randomized trial of patching regimens for treatment of moderate amblyopia in children. Arch. Ophthalmol. 121, 603–611. doi: 10.1001/archopht.121.5.603 12742836

[ref69] Rislove E. M. Hall E. C. Kiorpes L. (2005). Global form perception in human amblyopia studied using glass patterns. Invest. Ophthalmol. Vis. Sci. 46:3593.

[ref70] Rislove E. M. Hall E. C. Stavros K. A. Kiorpes L. (2010). Scale-dependent loss of global form perception in strabismic amblyopia. J. Vis. 10:25. doi: 10.1167/10.12.25 PMC3077296 21047757

[ref71] Shooner C. Hallum L. E. Kumbhani R. D. Ziemba C. M. Garcia-Marin V. Kelly J. G.. (2015). Population representation of visual information in areas V1 and V2 of amblyopic macaques. Vis. Res. 114, 56–67. doi: 10.1016/j.visres.2015.01.012 25637856 PMC4519437

[ref72] Stewart C. E. Moseley M. J. Fielder A. R. Stephens D. A. (2004). Refractive adaptation in amblyopia: quantification of effect and implications for practice. Br. J. Ophthalmol. 88, 1552–1556. doi: 10.1136/bjo.2004.044214 15548811 PMC1772452

[ref73] Tao X. Zhang B. Shen G. Wensveen J. Smith E. L. 3rd Nishimoto S.. (2014). Early monocular defocus disrupts the normal development of receptive-field structure in V2 neurons of macaque monkeys. J. Neurosci. 34, 13840–13854. doi: 10.1523/JNEUROSCI.1992-14.2014 25297110 PMC4188977

[ref74] Wallace D. K. Lazar E. L. Melia M. Birch E. E. Holmes J. M. Hopkins K. B.. (2011). Stereoacuity in children with anisometropic amblyopia. J. AAPOS 15, 455–461. doi: 10.1016/j.jaapos.2011.06.007 22108357 PMC3223370

[ref75] Wang Y. Zhang B. Tao X. Wensveen J. M. Smith E. L. R. Chino Y. M. (2017). Noisy spiking in visual area V2 of amblyopic monkeys. J. Neurosci. 37, 922–935. doi: 10.1523/JNEUROSCI.3178-16.2016 28123026 PMC5296785

[ref76] Wen W. Wang Y. Zhou J. He S. Sun X. Liu H.. (2021). Loss and enhancement of layer-selective signals in geniculostriate and corticotectal pathways of adult human amblyopia. Cell Rep. 37:110117. doi: 10.1016/j.celrep.2021.110117 34910903

[ref77] Zhang J. Y. Cong L. J. Klein S. A. Levi D. M. Yu C. (2014). Perceptual learning improves adult amblyopic vision through rule-based cognitive compensation. Invest. Ophthalmol. Vis. Sci. 55, 2020–2030. doi: 10.1167/iovs.13-13739 24550359 PMC3974581

